# Single center experience with first-intention high-frequency jet vs. volume-targeted ventilation in extremely preterm neonates

**DOI:** 10.3389/fped.2023.1326668

**Published:** 2024-01-04

**Authors:** Dimitrios Rallis, Danielle Ben-David, Kendra Woo, Jill Robinson, David Beadles, Laura Bernardini, Elisa Abdulhayoglu, Elizabeth Flanigan, Helen Christou

**Affiliations:** ^1^ Department of Pediatrics, Brigham and Women’s Hospital, Harvard Medical School, Boston, MA, United States; ^2^ Neonatal Intensive Care Unit, Faculty of Medicine, University of Ioannina, Ioannina, Greece

**Keywords:** bronchopulmonary dysplasia, infant, intubation, mechanical ventilation, respiratory distress syndrome

## Abstract

**Objectives:**

To examine whether first-intention high-frequency jet ventilation (HFVJ), compared to volume-targeted ventilation (VTV), in extremely preterm infants is associated with lower incidence of bronchopulmonary dysplasia (BPD) and other adverse clinical outcomes.

**Study design:**

We conducted a retrospective cohort study evaluating neonates with gestational age (GA) ≤28 weeks, who received first-intention HFJV (main exposure) or VTV (comparator), between 11/2020 and 3/2023, with a subgroup analysis including neonates with GA ≤26 weeks and oxygenation index (OI) >5.

**Results:**

We identified 117 extremely preterm neonates, 24 (GA 25.2 ± 1.6 weeks) on HFJV, and 93 (GA 26.4 ± 1.5 weeks, *p* = 0.001) on VTV. The neonates in the HFJV group had higher oxygenation indices on admission, higher inotrope use, and remained intubated for a longer period. Despite these differences, there were no statistically significant differences in rates of BPD, survival, or other adverse outcomes between the two groups. In subgroup analysis of 18 neonates on HFJV and 39 neonates on VTV, no differences were recorded in the GA, and duration of mechanical ventilation, while neonates in the HFJV group had significantly lower rates of BPD (50% compared to 83%, *p* = 0.034), and no significant differences in other adverse outcomes compared to neonates in the VTV group. In neonates ≤26 weeks of GA with OI >5, HFJV was significantly associated with lower rates of BPD (OR 0.21, 95% CI 0.05–0.92), and combined BPD or death (OR 0.18, 95% CI 0.03–0.85), after adjusting for birth weight, and Arterial-alveolar gradient on admission.

**Conclusions:**

In extremely preterm neonates ≤26 weeks of GA with OI >5, first-intention HFJV, in comparison to VTV, is associated with lower rates of BPD.

## Introduction

The introduction of antenatal steroids and surfactant replacement therapy has dramatically improved the survival rates of preterm neonates, including those born at or before 28 weeks' gestational age (GA) (1–3). However, extremely preterm neonates often require mechanical ventilation to achieve oxygenation and ventilation in the setting of respiratory distress syndrome (RDS) and/or respiratory failure (3, 4). Despite advances in the respiratory care of extremely preterm neonates, prolonged mechanical ventilation leads to ventilator-induced lung injury and can contribute to the development of bronchopulmonary dysplasia (BPD) (5–7). BPD is the most common morbidity among survivors of extreme prematurity (8, 9) and is associated with adverse neurodevelopmental outcomes and long-term pulmonary complications (10–12).

A protocolized initial ventilatory approach has been shown to improve outcomes in extremely preterm infants (13), however, the optimal initial (first-intention) strategy is not uniformly agreed upon (8, 13, 14). High-frequency jet ventilation (HFJV) has been used interchangeably with conventional ventilation and in some centers, as a first-intention ventilator approach (13). HFJV has the advantage that minute ventilation is achieved using tidal volumes smaller than physiologic dead space due to the use of high respiratory rates. Moreover, the rapid gas injection into the lungs produces flow streaming that sends gas via laminar and transitional flow. This results in lower delivered pressure to the alveoli, and thus reduces the risk of barotrauma and volutrauma (14, 15). Despite the theoretical advantage of HFJV over conventional ventilation and several reports supporting its use in extremely preterm neonates, there is no consensus on the use of HFJV compared to conventional ventilation as a first intention strategy in this vulnerable population. This is mainly because (i) earlier reports showed an association of HFJV with adverse neurological outcomes (16), and (ii) the introduction of conventional volume-targeted ventilation (VTV) in extremely preterm infants was shown to be effective in decreasing rates of BPD compared to pressure-limited ventilation (17).

In this study, we aimed to examine whether a standardized elective (first-intention) use of HFJV, compared to conventional VTV in extremely preterm neonates with RDS, would be associated with a lower incidence of BPD and other adverse clinical outcomes.

## Methods

We conducted a retrospective cohort study and included all mechanically ventilated extremely preterm (GA ≤28 weeks) neonates admitted to our neonatal intensive care unit in the Northeastern US, between 11/2020 and 3/2023. The study was approved by the institutional review board (Protocol Number 2023P001168/05.05.2023).

In our institution, thresholds for intubation in extremely preterm neonates include increased oxygen requirements [fraction of inspired oxygen (FiO_2_) >0.60], respiratory acidosis [pH <7.2 and partial pressure of carbon dioxide (PaCO_2_) >60 mmHg], or prolonged apnea, while surfactant is administrated when FiO_2_ >0.30 is required, or when intubation in the delivery room has been performed for primary resuscitation. Caffeine treatment is also recommended within the first 24 postnatal hours in all neonates <30 weeks GA, with the goal of starting caffeine within the first hour of life. The final decision of the selected first-intention mode was based on the preference of the providers during the study period. Historically, in our institution, VTV (assist-control/pressure regulated volume control) was the preferred initial ventilation strategy for extremely preterm neonates, and this is reflected in the early phase of the study period. Recently, HFJV gradually became the initial ventilation strategy initially due to limited availability of conventional ventilators during the COVID pandemic and subsequently due to a shift in our practice within our newly established Small Baby Program. The launch of the Small Baby Program emphasized the need for a standardized ventilator approach for these vulnerable infants with HFJV as the preferred initial ventilator mode. This decision was based on our acquired experience with HFJV, recognition of the theoretical advantages of HFJV, evidence from published studies, and discussions of institutional experience in other small baby programs such as the program at the University of Iowa (13, 15). The initial settings on VTV (Drägerwerk AG & Co, Lübeck, Germany) were tidal volume 5–6 ml/kg, positive end-expiratory pressure (PEEP) 6 cmH_2_O, pressure max 26–28 cmH_2_O, inspiratory time 0.3–0.4 s and back-up rate 30–35/min, whereas when first-intention HFJV mode [Model 203 Life Pulse High-Frequency Ventilator (Jet), Bunnell, INC, Salt Lake City, UT, US] was utilized, the initial settings were rate: 300–360/min, inspiratory time: 0.02 s, peak inspiratory pressure: 20–24 cmH_2_O, PEEP: 6 cmH_2_O, with no backup rate. Neonates that stayed on the selected first-intention mode for more than an hour qualified to be included in the study.

We collected the perinatal characteristics, and clinical data during the initial mode of ventilation including MAP, FiO_2_, pH, partial pressure of oxygen (PaO_2_), PaCO_2_, oxygenation index (OI), Alveolar-arterial gradient, and arterial/Alveolar ratio for the two groups. Respiratory severity indices were calculated, as follows: OI = [FiO2*(MAP)*100]/PaO_2_, Alveolar-arterial gradient = [[FiO_2_*(Patm-PH_2_O)]-(PaCO_2_/R)]-PaO_2_, and arterial/Alveolar ratio = PaO_2_/[[FiO_2_*(Patm-PH_2_O)]-(PaCO_2_/R)]. The primary outcome of our study was comparison of BPD rates between neonates in the HFJV and VTV groups. BPD was defined according to the 2018 criteria of the National Institute of Child Health and Development, by the existence of persistent parenchymal lung disease, radiographic confirmation of parenchymal lung disease, and the need for oxygen administration for ≥3 consecutive days to maintain arterial oxygen saturation in the 90%–95% range, at 36 weeks of corrected age (18).

Moreover, to evaluate the differences in BPD rates between neonates ≤26 weeks of GA with significant RDS in the HFJV and VTV groups, we performed a subgroup analysis including only neonates ≤26 weeks of GA with an initial OI >5. These were arbitrary thresholds used retroactively to define severity of RDS within our group and did not influence clinical decision-making pertaining to the selection of the first-intention mode of ventilation. Secondary outcomes included the duration of invasive mechanical ventilation, rates of combined BPD or death, other common neonatal morbidities [early and late-onset sepsis, intraventricular hemorrhage, periventricular leukomalacia, retinopathy of prematurity, necrotizing enterocolitis based on Vermont—Oxford Network criteria (19)], and survival. All patients' outcomes were tracked to hospital discharge regardless of transfer.

### Statistical analysis

Continuous variables were expressed as mean ± standard deviation or median (interquartile range), and categorical variables as *n* (percentage %). The normality of the distributions of continuous variables was assessed by the Kolmogorov–Smirnov or the Shapiro–Wilk test. Comparisons between continuous variables were performed with the student's *t*-test, or the non-parametric Mann–Whitney test, as appropriate, whereas comparisons between categorical variables utilizing the chi-square test or the Fisher's exact test.

A multivariate logistic regression analysis was used to evaluate the effect of the first-intention HFJV mode in neonates ≤28 weeks of GA (independent variable) on BPD, or combined BPD or death (dependent variables), adjusted for GA, birth weight, and OI on admission. Also, the multivariate logistic regression model was used to evaluate the effect of the first-intention HFJV mode in neonates ≤26 weeks of GA with OI >5 (independent variable) on BPD, or combined BPD or death (dependent variables), adjusted for birth weight, and Arterial-alveolar gradient on admission. Among the factors examined, only those with a significant effect in univariate analysis were included in the multivariate model. Odds ratios (OR) and 95% confidence intervals (CI) were calculated. All performed tests were two-sided and a *p*-value less than 0.05 was considered statistically significant (alpha 0.05). The data were analyzed using SPSS Statistics Version 25.0 (IBM SPSS Statistics for Windows, Version 24.0. Armonk, NY, US).

## Results

**Figure 1:**
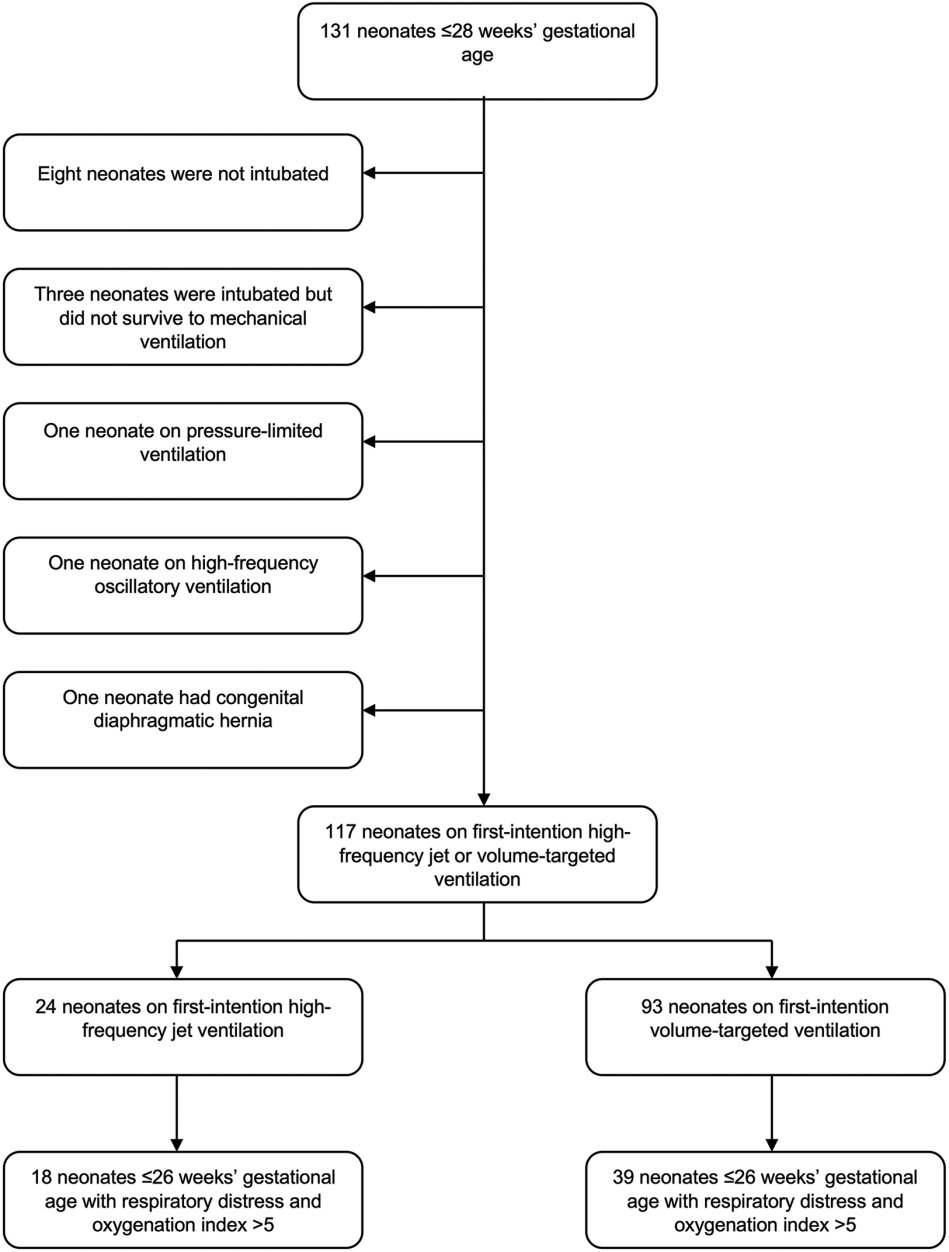
Flowsheet of the study population.

One hundred thirty-one neonates ≤28 weeks were admitted to our center in the study period. Fourteen neonates were excluded as ineligible [eight neonates who were not intubated, three neonates who were intubated but did not survive to initiation of mechanical ventilation, two neonates who were started on pressure-limited ventilation or high-frequency oscillatory ventilation (one each), and one neonate with congenital diaphragmatic hernia (Figure 1)], and 117 neonates were analyzed, of which 24 were started on first-intention HFJV and 93 on VTV. Neonates of the HFJV compared to the VTV group were of a significantly lower GA (25.2 ± 1.6 compared to 26.4 ± 1.5 weeks, *p* = 0.001) and birth weight (726 ± 164 compared to 910 ± 255 g, *p* = 0.001) (Table 1), and also, they had significantly higher oxygenation indices on their first recorded blood gas (at 1.5 h of life for the HFJV group and at 2 h of life for the VTV group) (Table 2). Both first-intention HFJV and VTV modes were started by 1 h after birth. First-intention HFJV mode was applied uninterrupted for 2.5 (0.8–17.1) days while VTV mode for 0.6 (0.2–1.1) days (*p* < 0.001) (Table 3). Moreover, neonates of the HFJV group ventilated for a significantly longer period [15 (5–30) compared to 4 (1–18) days, *p* = 0.021], and received inotropes (54% compared to 22%, *p* = 0.004) and opioids at higher rates compared to the VTV group (87% compared to 62%, *p* = 0.026). Of note, 39% of neonates in the VTV group were ventilated with a high-frequency mode (“rescue” high-frequency ventilation), and 68% of neonates in the HFJV group with VTV during their hospital stay (Table 3). Besides the above differences, there were no significant differences in the rates of BPD between neonates of the HFJV compared to the VTV group. Similarly, there were no significant differences in the rates of late-onset sepsis, intraventricular hemorrhage, periventricular leukomalacia, retinopathy of prematurity, survival, or the combined outcome of BPD or death between the two groups (Table 3).

**Table 1:** Perinatal characteristics of the study population.

	HFJV (*n* = 24)	VTV (*n* = 93)	*p*
Gestational age, weeks	25.2 ± 1.6	26.4 ± 1.5	0.001
Birthweight, g	726 ± 164	910 ± 255	0.001
Sex, male	13 (54%)	50 (54%)	1.000
Intrauterine growth restriction	3 (13%)	14 (15%)	1.000
Delivery mode, cesarean section	17 (71%)	72 (77%)	0.592
Prolonged rupture of membranes	14 (58%)	36 (39%)	0.106
Maternal group B *Streptococcus* colonization	3 (13%)	8 (9%)	0.694
Chorioamnionitis	6 (25%)	11 (12%)	0.114
Antenatal steroids			0.189
Complete	17 (71%)	47 (51%)	
Incomplete	4 (17%)	31 (33%)	
Apgar 1st minute	4 (2–6)	4 (2–6)	0.833
Apgar 5th minute	7 (6–8)	7 (6–8)	0.925
Intubation in the delivery room	21 (88%)	68 (73%)	0.184
Surfactant doses	2 (2–2)	2 (1–2)	0.648
Caffeine initiation, hours	2 (1–3)	2 (2–3)	0.994

HFJV, high-frequency jet ventilation; VTV, volume-targeted ventilation.

Continuous variables are expressed as mean ± SD or median (interquartile range). *P*-values of student's *t*-test or Mann–Whitney test. Categorical variables are expressed as *n* (%). *P*-values of chi-square test or Fisher's exact test.

**Table 2:** Ventilator settings and blood gas characteristics of the study cohort on admission.

	HFJV (*n* = 24)	VTV (*n* = 93)	*p*
Time after birth for first blood gas, hours	1.5 (1–2)	2 (1–2)	0.276
Mean airway pressure, cmH_2_O	10 (8–11)	10 (9–11)	0.872
FiO_2_	0.44 (0.34–0.69)	0.30 (0.21–0.35)	<0.001
pO_2_, mmHg	49 (44–65)	45 (38–56)	0.114
pCO_2_, mmHg	47 (41–54)	45 (39–50)	0.582
pH	7.27 (7.22–7.33)	7.32 (7.25–7.36)	0.112
Oxygenation index	8 (7–11)	6 (4–7)	<0.001
Oxygenation index >5	22 (92%)	66 (70%)	0.035
Arterial-alveolar gradient, mmHg	197 (134–361)	105 (55–143)	<0.001
Alveolar/arterial ratio	0.19 (0.13–0.23)	0.31 (0.24–0.44)	<0.001

HFJV, high-frequency jet ventilation; VTV, volume-targeted ventilation; FiO_2_, fraction of inspired oxygen; pO_2_, partial pressure of oxygen; pCO_2_ partial pressure of carbon dioxide.

Continuous variables are expressed as mean ± SD or median (interquartile range). *P*-values of student's *t*-test or Mann–Whitney test. Categorical variables are expressed as *n* (%). *P*-values of chi-square test or Fisher's exact test.

**Table 3:** Clinical care needs and outcomes of the study population.

	HFJV (*n* = 24)	VTV (*n* = 93)	*p*
Age when first-intention mode was initiated, hours of life	1 (1–1)	1 (1–1.5)	0.219
Duration of uninterrupted first-intention mode, days	2.5 (0.8–17.1)	0.6 (0.2–1.1)	<0.001
Mechanical ventilation duration, days	15 (5–30)	4 (1–18)	0.021
Ventilated at 72 h of life	19 (86%)	45 (51%)	0.003
Ventilated at seven days of life	15 (68%)	37 (42%)	0.033
Any high frequency ventilation during course	24 (100%)	36 (39%)	<0.001
Any conventional ventilation during course	13 (68%)	93 (100%)	0.004
Non-invasive mechanical ventilation duration, days	34 (22–40)	39 (28–43)	0.096
Pneumothorax	1 (4%)	10 (11%)	0.456
Pulmonary hemorrhage	1 (4%)	7 (8%)	1.000
Early-onset sepsis	–	1 (1%)	1.000
Intraventricular hemorrhage			0.627
No	13 (54%)	46 (50%)	
I	2 (8%)	19 (21%)	
II	4 (17%)	12 (13%)	
III	2 (8%)	6 (7%)	
IV	3 (13%)	8 (9%)	
Postnatal steroids	8 (33%)	27 (29%)	0.803
Opioids	21 (87%)	58 (62%)	0.026
Opioids duration, days	8 (2–30)	6 (2–23)	0.652
Paralysis	3 (13%)	14 (15%)	1.000
Paralysis duration, days	1 (1–2)	2 (1–5)	0.155
Sedation	3 (12%)	8 (9%)	0.694
Sedation duration, days	1 (1–2)	5 (1–30)	0.279
Inotropes	13 (54%)	20 (22%)	0.004
Patent ductus arteriosus	17 (71%)	56 (60%)	0.479
Necrotizing enterocolitis	3 (13%)	7 (8%)	0.426
Late-onset sepsis	11 (46%)	32 (35%)	0.352
Periventricular leukomalacia	1 (4%)	4 (4%)	1.000
Retinopathy of prematurity	9 (50%)	45 (50%)	1.000
Bronchopulmonary dysplasia	11 (55%)	55 (66%)	0.442
Bronchopulmonary dysplasia grade			0.465
No	9 (45%)	29 (35%)	
I	4 (20%)	30 (36%)	
II	4 (20%)	18 (21%)	
III	3 (15%)	7 (8%)	
Length of stay, days	96 (32–111)	98 (75–115)	0.513
Survival	19 (79)	80 (86%)	0.525
Combined bronchopulmonary dysplasia or death	16 (67%)	65 (70%)	0.806

HFJV, high-frequency jet ventilation; VTV, volume-targeted ventilation.

Continuous variables are expressed as mean ± SD or median (interquartile range). *P*-values of student's *t*-test or Mann–Whitney test. Categorical variables are expressed as *n* (%). *P*-values of chi-square test or Fisher's exact test.

In multivariate regression analysis in neonates ≤28 weeks of GA, HFJV mode was significantly associated with lower rates of combined BPD or death (OR 0.28, 95% CI 0.09–0.94, *p* = 0.040), after adjusting for GA, birth weight, and OI on admission (Table 4).

**Table 4:** Multivariate regression analysis of the association of HFJV with bronchopulmonary dysplasia, and with combined bronchopulmonary dysplasia or death, adjusted for gestational age, birth weight, and oxygenation index, in extremely preterm neonates ≤28 weeks of gestational age with respiratory distress syndrome.

	OR	95% CI	*p*
Bronchopulmonary dysplasia			
First intention HFJV	0.36	0.12–1.11	0.077
Gestational age	0.91	0.82–1.03	0.058
Birth weight	0.99	0.95–0.99	0.008
Oxygenation index on admission	1.01	0.90–1.13	0.836
Combined bronchopulmonary dysplasia or death			
First intention HFJV	0.28	0.09–0.94	0.040
Gestational age	0.94	0.86–1.04	0.292
Birth weight	0.99	0.95–0.99	0.004
Oxygenation index on admission	1.20	1.02–1.42	0.025

HFJV, high-frequency jet ventilation; OR, odds ratio; CI, confidence intervals.

Among the factors examined, only those with a significant effect in univariate analysis, with a *p*-value cut-off value <0.05, were included in the multivariate model.

Within this extremely preterm cohort of neonates ≤26 weeks of GA, 57 neonates had OI >5 (Figure 1); 18 neonates in the HFJV group with a mean GA of 24.6 ± 1.2 weeks and birth weight 715 ± 151 g, and 39 neonates in the VTV group with a mean GA of 25.2 ± 0.9 weeks and birth weight 755 ± 165 g (Table 5). For this subgroup of sicker neonates, ventilator settings and blood gas characteristics on first measurement (within 1 h after birth for HFJV and within 1.5 h for VTV) were not significantly different between the two groups, except for a significantly higher Alveolar-arterial gradient in the HFJV group compared to that of the VTV group (Table 6). Both first-intention HFJV and VTV modes were started by one hour after birth, and first-intention HFJV mode was applied uninterrupted for 3.8 (0.9–18.4) days while VTV mode for 0.8 (0.6–1.8) days (*p* = 0.001) (Table 7). Within this same group of patients, 22% of neonates in the VTV group ventilated with high-frequency ventilation and 77% of neonates in the HFJV group with VTV during their hospital stay (Table 7). In this subgroup analysis, neonates in the HFJV compared to the VTV group had significantly lower rates of BPD (50% compared to 83%, *p* = 0.034). Furthermore, there were no significant differences in the rates of late-onset sepsis, intraventricular hemorrhage, periventricular leukomalacia, retinopathy of prematurity, survival, or the combined outcome of BPD or death between the two groups (Table 7).

**Table 5:** Subgroup analysis of perinatal characteristics for neonates ≤26 weeks of gestational age with respiratory distress syndrome and oxygenation index >5.

	HFJV (*n* = 18)	VTV (*n* = 39)	*p*
Gestational age, weeks	24.6 ± 1.2	25.2 ± 0.9	0.138
Birthweight, g	715 ± 151	755 ± 165	0.377
Sex, male	8 (44%)	23 (59%)	0.394
Intrauterine growth restriction	2 (11%)	6 (15%)	1.000
Delivery mode, cesarean section	12 (67%)	29 (74%)	0.752
Prolonged rupture of membranes	10 (56%)	16 (41%)	0.394
Maternal Group B *Streptococcus* colonization	2 (11%)	3 (8%)	0.646
Chorioamnionitis	4 (22%)	6 (15%)	0.709
Antenatal steroids			0.447
Complete	12 (67%)	18 (46%)	
Incomplete	3 (17%)	11 (28%)	
Apgar 1st minute	4 (1–5)	4 (2–5)	0.599
Apgar 5th minute	7 (5–8)	7 (5–7)	0.855
Intubation in the delivery room	16 (89%)	32 (82%)	0.704
Surfactant doses	2 (2–2)	2 (2–2)	0.967
Caffeine initiation, hours	2 (1–2)	2 (1–3)	0.552

HFJV, high-frequency jet ventilation; VTV, volume-targeted ventilation.

Continuous variables are expressed as mean ± SD or median (IQR). *P*-values of student's *t*-test or Mann–Whitney test. Categorical variables are expressed as *n* (%). *P*-values of chi-square test or Fisher's exact test.

**Table 6:** Subgroup analysis of the ventilator settings and blood gas characteristics of neonates ≤26 weeks of gestational age with respiratory distress syndrome and oxygenation index >5 on admission.

	HFJV (*n* = 18)	VTV (*n* = 39)	*P*
Time after birth for first blood gas, hours	1 (1–2)	1.5 (1–2)	0.539
Mean airway pressure, cmH_2_O	10 (9–11)	10 (9–11)	0.909
FiO_2_	0.45 (0.45–0.68)	0.34 (0.30–0.51)	0.018
pO_2_, mmHg	49 (44–56)	45 (40–55)	0.289
pCO_2_, mmHg	47 (40–56)	45 (42–53)	0.973
pH	7.25 (7.22–7.33)	7.29 (7.22–7.34)	0.481
Oxygenation index	9 (7–13)	8 (6–10)	0.053
Arterial-alveolar gradient, mmHg	211 (184–366)	142 (112–242)	0.029
Alveolar/arterial ratio	0.17 (0.12–0.21)	0.24 (0.16–0.29)	0.050

HFJV, high-frequency jet ventilation; VTV, volume-targeted ventilation; FiO_2_, fraction of inspired oxygen; pO_2_, partial pressure of oxygen; pCO_2_, partial pressure of carbon dioxide.

Continuous variables are expressed as mean ± SD or median (IQR). *P*-values of student's *t*-test or Mann–Whitney test.

**Table 7:** Subgroup analysis of the clinical care needs and outcomes of neonates ≤26 weeks of gestational age with respiratory distress syndrome and oxygenation index >5.

	HFJV (*n* = 18)	VTV (*n* = 39)	*p*
Age when first-intention mode was initiated, hours of life	1 (1–1)	1 (1–1)	0.531
Duration of uninterrupted first-intention mode, days	3.8 (0.9–18.4)	0.8 (0.6–1.8)	0.001
Mechanical ventilation duration, days	18 (9–27)	18 (3–43)	0.536
Ventilated at 72 h of life	15 (94%)	26 (72%)	0.140
Ventilated at seven days of life	12 (75%)	24 (67%)	0.747
Any high frequency ventilation during course	18 (100%)	22 (56%)	<0.001
Any conventional ventilation during course	10 (77%)	39 (100%)	0.277
Non-invasive mechanical ventilation duration, days	35 (21–42)	30 (24–41)	0.978
Pneumothorax	1 (6%)	7 (18%)	0.414
Pulmonary hemorrhage	1 (6%)	4 (10%)	1.000
Early-onset sepsis	–	1 (1%)	1.000
Intraventricular hemorrhage			0.397
No	9 (50%)	14 (37%)	
I	–	6 (16%)	
II	4 (22%)	7 (18%)	
III	3 (17%)	4 (11%)	
IV	2 (11%)	7 (18%)	
Postnatal steroids	5 (28%)	23 (59%)	0.045
Opioids	17 (94%)	29 (74%)	0.146
Opioids duration, days	8 (2–30)	23 (4–43)	0.103
Paralysis	2 (11%)	10 (26%)	0.303
Paralysis duration, days	3 (2–4)	5 (3–9)	0.364
Sedation	2 (11%)	5 (13%)	1.000
Sedation duration, days	1 (1–2)	8 (2–10)	0.190
Inotropes	10 (56%)	15 (39%)	0.262
Patent ductus arteriosus	13 (72%)	30 (77%)	0.747
Necrotizing enterocolitis	2 (11%)	6 (15%)	1.000
Late-onset sepsis	9 (50%)	20 (53%)	1.000
Periventricular leukomalacia	–	1 (3%)	1.000
Retinopathy of prematurity	7 (58%)	25 (68%)	0.729
Bronchopulmonary dysplasia	7 (50%)	28 (83%)	0.034
Bronchopulmonary dysplasia grade			0.177
No	7 (50%)	6 (18%)	
I	4 (29%)	13 (38%)	
II	2 (14%)	9 (26%)	
III	1 (7%)	6 (18%)	
Length of stay, days	88 (80–110)	111 (80–136)	0.099
Survival	13 (72%)	30 (77%)	0.747
Combined bronchopulmonary dysplasia or death	12 (67%)	34 (87%)	0.068

HFJV, high-frequency jet ventilation; VTV, volume-targeted ventilation.

Continuous variables are expressed as mean ± SD or median (IQR). *P*-values of student's *t*-test or Mann–Whitney test. Categorical variables are expressed as *n* (%). *P*-values of chi-square test or Fisher's exact test.

In multivariate regression analysis including neonates ≤26 weeks of GA with OI >5, HFJV mode was significantly associated with lower rates of BPD (OR 0.21, 95% CI 0.05–0.92, *p* = 0.039), and combined BPD or death (OR 0.18, 95% CI 0.03–0.85, *p* = 0.031), after adjusting for birth weight, and Arterial-alveolar gradient on admission (Table 8).

**Table 8:** Multivariate regression analysis of the association of HFJV with bronchopulmonary dysplasia, and with combined bronchopulmonary dysplasia or death, adjusted for gestational age, birth weight, and arterial-alveolar gradient on admission, in extremely preterm neonates ≤26 weeks of gestational age with respiratory distress syndrome and oxygenation index >5.

	OR	95% CI	*p*
Bronchopulmonary dysplasia			
First intention HFJV	0.21	0.05–0.92	0.039
Birth weight	0.99	0.91–1.02	0.233
Arterial-alveolar gradient on admission	1.04	0.96–1.01	0.111
Combined bronchopulmonary dysplasia or death			
First intention HFJV	0.18	0.03–0.85	0.031
Birth weight	0.96	0.90–1.01	0.083
Arterial-alveolar gradient on admission	1.02	0.97–1.08	0.428

HFJV, high-frequency jet ventilation; OR, odds ratio; CI, confidence intervals.

Among the factors examined, only those with a significant effect in univariate analysis, with a *p*-value cut-off value <0.05, were included in the multivariate model.

## Discussion

Extremely preterm neonates treated with a standardized first-intention HFJV compared to neonates treated with VTV had similar rates of BPD or combined BPD or death, despite being of lower GA and birth weight, having higher oxygenation indices and receiving inotropes at higher rates. Interestingly, in a subgroup analysis of extremely preterm neonates ≤26 weeks of GA with OI >5, neonates receiving first-intention HFJV compared to neonates receiving VTV had significantly lower rates of BPD. Among neonates ≤26 weeks of GA with OI >5, HFJV was significantly associated with lower rates of BPD and combined BPD or death, after adjusting for birth weight, and Arterial-alveolar gradient on admission.

Our findings are consistent with previously reported evidence supporting the potential benefit of first-intention HFJV over conventional ventilation in extremely preterm infants with RDS (20, 21). It should be noted that the previous studies (20, 21) were performed during an era when there was significantly different approaches on the ventilation management of extremely preterm neonates compared to our cohort; however, there is a strong physiologic rationale for use of first-intention HFJV, which is based on the stage of fetal lung development at the time of birth. The extremely preterm lung is exceptionally vulnerable to volutrauma and shearing injury, which is minimized by use of jet tidal volumes smaller than physiologic dead space and the ability to effectively ventilate and oxygenate at lower mean airway pressures compared to conventional ventilation (13, 15). The avoidance of large tidal volumes is lung protective and reduces secondary lung injury and subsequent development of BPD. It also decreases like risk of hyperinflation and air leak syndromes, which have been shown to increase morbidity and mortality in extremely preterm infants (13, 15). The tidal volumes delivered by HFJV are limited, minimizing the risk of volutrauma and oxygen toxicity, which contribute to lung injury and the development of BPD (14, 15). In our study, when we examined neonates with more severe RDS (OI >5), we found that those in the HFJV group had lower rates of BPD, or BPD/death, compared to neonates of the VTV group. Previously Keszler et al., in a multicenter randomized clinical trial where both high- and low-volume strategies were used (of note, the high-volume strategy was used by protocol with only a modest proportion of infants treated with the low-volume approach in violation of the protocol), demonstrated a significant reduction in BPD in preterm neonates with RDS who received surfactant and were initially on HFJV compared to those on conventional ventilation (21). In contrast, Wiswell et al. reported no difference in BPD rates in a randomized controlled trial of elective HFJV utilizing a low-volume strategy compared to neonates on conventional ventilation (16). A Cochrane meta-analysis by Bhuta and Henderson-Smart (22) including the above two studies and the randomized trial by Carlo et al. (23) who also used a low MAP strategy, concluded that the elective use of HFJV for preterm infants with RDS was associated with an overall reduction in the rate of BPD at 36 weeks corrected age (relative risk 0.58, 95% CI 0.34–0.98).

A relative skepticism, however, persists due to the reported association of first-intention HFJV with significant adverse effects including intraventricular hemorrhage, cystic periventricular leukomalacia, and death in extremely preterm neonates (16). We found no differences in adverse respiratory or non-respiratory outcomes between the first intention HFJV and VTV groups, even within the subgroup of sicker neonates. Specifically, we found no differences in rates of pneumothorax or pulmonary hemorrhage between the two groups. Neonates in the HFJV group were intubated for a longer period and received opioids at higher rates compared to those in the VTV group. Regarding the non-respiratory adverse outcomes, we found no differences between the HFJV and VTV groups in intraventricular hemorrhage, periventricular leukomalacia, retinopathy of prematurity, necrotizing enterocolitis, and early or late onset sepsis. Previously Wiswell et al. reported an increase in adverse outcomes such as cystic periventricular leukomalacia and/or death in a group of infants who received first-intention HFJV compared to those receiving conventional ventilation in a randomized controlled trial (16). The same group also examined the effect of hypotension, acidosis, hypoxemia, and hypocarbia during the first 3 days of life on the development of periventricular leukomalacia in premature neonates ventilated with HFJV, revealing that infants with cystic periventricular leukomalacia were independently significantly more likely to have greater cumulative hypocarbia below a threshold level of 25 mm Hg during the first day of life (24). Furthermore, similar trials conducted by Keszler et al. (21) and Carlo et al. (23), each found no significant increase in non-pulmonary morbidities for neonates in the HFJV compared to the conventional ventilation group. It is plausible that differences in ventilator strategies between Wiswell's (low-volume strategy) and Keszler's (high-volume strategy) studies, including the use of markedly different initial PEEP, as well as different approaches to the management of ventilation and the targeted PaCO_2_ levels, might have contributed to the different outcomes reported (16, 21). Overall, the meta-analysis by Bhuta and Henderson-Smart concluded that there were no significant differences in mortality, overall incidence of any intraventricular hemorrhage or severe intraventricular hemorrhage, or air leaks (22). The final conclusion of the meta-analysis is that more research is needed before any recommendation favoring HFJV as a primary mode over conventional ventilation in preterm infants could be made (22). This is due to the limited evidence from randomized trials and the uncertainty regarding adverse effects associated with HFJV.

In our study, a significant proportion of neonates in the VTV group received rescue high-frequency ventilation (either jet or oscillatory). Similarly, a relatively high proportion of neonates in the HFJV group received VTV (68%). This is an important limitation of our study that does not allow us to examine the effect of HFJV in the later phases of respiratory disease when alveolar and airway injury have already occurred. When HFJV was first introduced in the pre-surfactant era and prior to advances in VTV its main use was as a lung protective strategy when conventional ventilation failed, i.e., settings on conventional ventilation escalated to levels that were deemed unsafe. This “rescue” strategy was proven effective in prior reports (25–29) even for moribund neonates with air leaks in which HFJV improved survival (26). More recently, Plavka et al. reported that, compared with conventional ventilation or high-frequency oscillatory ventilation, HFJV improved gas exchange and facilitated weaning from mechanical ventilation in extremely preterm neonates with evolving BPD (27). A Cochrane review completed in 2006 (30) and updated in 2015 (31), concluded that existing evidence in support of HFJV as a rescue strategy in preterm infants was of low quality and thus this practice could not be supported.

Our study has several limitations. First, our findings from a single-center cohort study may not be generalizable. Moreover, this was a retrospective study, and although we collected a large number of variables, we acknowledge that outcomes such as BPD are multifactorial and that we were unable to examine additional variables that could potentially contribute to the outcomes of interest. Also, as per the study design, we could not examine the effect of any ventilation strategies beyond the initial first-intention approach on the outcomes evaluated. We also acknowledge the significant proportion of neonates who crossed over between the two groups, reflecting the inevitable limitations within clinical practice.

## Conclusion

This study supports that extremely preterm neonates (GA ≤28 weeks) treated with a standardized first-intention HFJV compared to neonates treated with VTV had similar rates of BPD, combined BPD or death, and other adverse outcomes. Within this group, preterm neonates ≤26 weeks of GA with RDS and OI >5 who were treated with first-intention HFJV compared to those treated with VTV had significantly lower rates of BPD, and no significant differences in other adverse outcomes. HFJV was significantly associated with lower rates of BPD, and combined BPD or death, after adjusting for birth weight, and OI on admission. Further prospectively designed studies are warranted to examine the effects of first-intention HFJV on limiting lung injury, improving survival, and optimizing neurodevelopmental outcomes.

## Data Availability

The original contributions presented in the study are included in the article/Supplementary Materials, further inquiries can be directed to the corresponding author.
